# Characteristics of coronary artery ectasia and accompanying plaques: an optical coherence tomography study

**DOI:** 10.1007/s10554-023-02835-9

**Published:** 2023-04-26

**Authors:** Huai Yu, Jiannan Dai, Hao Tang, Chao Fang, Senqing Jiang, Xueming Xu, Bo Yu, Yingfeng Tu

**Affiliations:** 1 Department of Cardiology, The Second Affiliated Hospital of Harbin Medical University, Harbin, China; 2 The Key Laboratory of Myocardial Ischemia, Chinese Ministry of Education, Harbin, China; 3 The First Affiliated Hospital of Harbin Medical University, Harbin, China

**Keywords:** Coronary artery ectasia, Optical coherence tomography, Atherosclerotic plaques, ST-segment elevation myocardial infarction, Vulnerable plaque

## Abstract

Coronary artery ectasia (CAE) in adults is often caused by atherosclerotic plaques. CAE can affect atherosclerotic plaques through hemodynamic changes. However, no study has evaluated the characteristics of CAE with atherosclerotic plaques. Therefore, we aimed to disclose the characteristics of atherosclerotic plaques in patients with CAE using optical coherence tomography (OCT). We evaluated patients with CAE, confirmed by coronary angiography, who underwent pre-intervention OCT between April 2015 and April 2021. Each millimeter of the OCT images was analyzed to assess the characteristics of CAEs, plaque phenotypes, and plaque vulnerability. A total of 286 patients (344 coronary vessels) met our criteria, 82.87% of whom were men. Right coronary artery lesions were the most common, comprising 44.48% (n = 153) of the total. We found 329 CAE vessels with plaques, accounting for 95.64% of the coronary vessels. After grouping CAEs and plaques by their relative positions, we found that the length of plaques within CAE lesions was longer than that of plaques in other sites (P < 0.001). Plaques within CAE lesions had greater maximum lipid angles and lipid indexes (P = 0.007, P = 0.004, respectively) than those on other sites. This study revealed the most common vascular and morphological characteristics of CAE. While the accompanying plaques were not affected by the location or morphology of the CAE vessels, they were affected by their position relative to the CAE lesion.

**Supplementary Information:**

The online version contains supplementary material available at 10.1007/s10554-023-02835-9.

## Introduction

Coronary artery ectasia (CAE) is classically defined as the dilation of a coronary artery segment to a diameter at least 1.5 times that of the adjacent non-dilated segments. The estimated prevalence of CAE is variable between studies and ranges between 1.2 and 7.4%, with a male predominance of about 90% [1, 2]. The two most common etiologies of CAE are atherosclerosis (AS), which accounts for approximately 50% of cases, and Kawasaki Disease (KD) [3]. The abnormal vessel dilatation of CAE is associated with disturbed coronary blood flow [4], which tends to increase the vulnerability of atherosclerotic plaques. CAE leads to hemodynamic disturbances, which can have many effects, including endothelial shear stress (ESS) etc., a condition that affects plaque vulnerability [5]. Accordingly, CAE has been associated with a 3.25-fold increase in the risk of major cardiac events, including cardiac death and non-fatal myocardial infarction (MI) [6]. The reported incidence of CAE-associated events, such as thrombosis, distal embolization, rupture, and vasospasm, varies from 1.5 to 5% [7].

However, few studies have evaluated the relationship between CAE and atherosclerotic plaques. Therefore, the objectives of this study were to map CAEs and intravascular plaques and analyze the features of CAE-associated plaques using optical coherence tomography (OCT) in vivo. This study provides insight into potential treatment options for reducing plaque vulnerability and the risk of major cardiac events in patients with CAE.

## Methods

### Study population

**Fig. 1:**
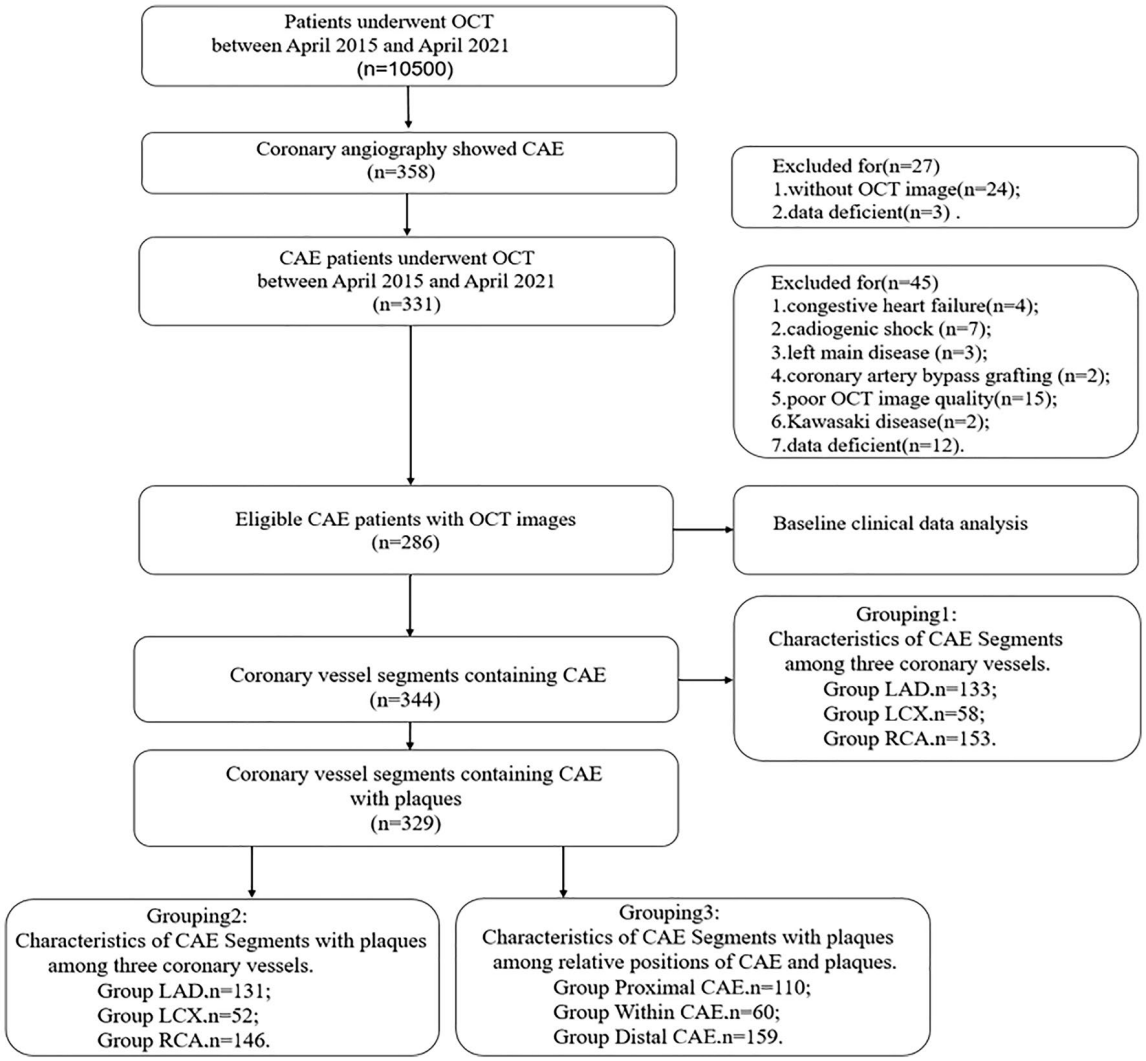
Flow chart

This study was a single-center retrospective study. Between April 2015 and April 2021, 358 coronary angiographies were screened for CAE using the OCT database at the 2nd Affiliated Hospital of Harbin Medical University. Based on the established angiographic and OCT standards, 331 patients (408 coronary vessels) were diagnosed with CAE and enrolled for further analysis. The main exclusion criteria were congestive heart failure, cardiogenic shock, left main disease, coronary artery bypass grafting, poor OCT image quality, extremely tortuous, heavily calcified vessels and KD were excluded from the study. Atherosclerosis seems to be the most frequent cause in adults [8], while KD, a systemic vasculitis with coronary tropism, is the most common etiology of CAE in childhood [9]. According to previous study, patients with KD-associated CAE have distinctive pathological features, such as the preferential involvement of proximal rather than distal segments and the development of focal rather than diffuse coronary dilatations [10]. Moreover, all included patients were adults and had no other physical problems or anything else untoward of KD in their medical history. Finally, 344 coronary vessels containing CAE from 286 patients were included in the study (Fig. 1). This study was approved by the Institutional Review Board participating site. Additional details on methods are included in the Supplementary material online.

### OCT image acquisition

Frequency-domain OCT C7-XR (Light lab Imaging Inc./St Jude Medical, Inc., Westford, MA) using the ILUMIEN OCT system (Abbott Vascular, Santa Clara, CA, USA) and the Dragon Fly catheter (Lightlab Imaging/St. Jude Medical, Westford, Massachusetts, USA) were used to acquire the OCT images. With the C7-XR system, we carefully advanced a 2.7 F OCT imaging catheter into the distal part of the culprit lesion. Automated pullback began when the blood was displaced by a brief injection of contrast agents or Dextran through the guiding catheter. The images were digitally stored for off-line analysis. The culprit vessel was identified based on clinical, electrocardiographic, and angiographic data. Prior to OCT imaging, aspiration thrombectomy was used for patients with thrombolysis in myocardial infarction (TIMI_ flow grade ≤ 2, but balloon catheter predilation was not used.

We acknowledge that the application of OCT in CAE is limited by incomplete blood clearance and low penetration depth in large vessels. To analyze the dilated coronary arteries, OCT was reconstructed using a “zoom-out” function to change the zoom level of field-of-view (FOV), which allows a more extensive assessment of lumen structures. And it enables up to 9.5 mm diameters of CAE in coronary artery segments from OCT imaging analysis. Moreover, the maximum diameter of the coronary artery was 8.64 mm in this study to improve the accuracy of the imaging analysis.

All OCT images were analyzed using offline software (Light-Lab Imaging, Inc./St Jude Medical, Inc.) at the OCT Core Laboratory of the 2nd Hospital of Harbin Medical University.

### OCT definition and classification

For OCT analysis, we selected plaques in the smallest area of the lumen in the affected blood vessel segment because these plaques are most likely to have adverse effects on patients with CAE. In addition, these plaques were most characteristic of AS and were therefore most likely to affect the characteristics of CAE, given that AS can cause CAE.

The following definitions were used to assess the OCT images. Plaque rupture is considered an intimal interruption and cavity formation in the plaque. Plaque erosion was defined as the presence of an attached thrombus overlying an intact and visualized plaque without fibrous cap disruption or cavity rupture. Calcium referred to well-delineated, signal-poor regions with sharp borders. Calcified nodules (CN) were defined as calcified plaques characterized by protruding, superficial calcium. A lipid-rich plaque was defined as a lipid plaque with lipid angles exceeding 90° on any cross-sectional image of the plaque. A thin-cap fibroatheroma (TCFA) was defined as a lipid-rich plaque with a fibrous cap ≤ 65 μm thick. Macrophages, microchannels, and cholesterol crystals were designated according to the established criteria for OCT. The lipid index was calculated as the product of the lipid core length and the mean lipid core angle [11].

The minimal lumen diameter (MLD) was the diameter of the smallest lumen in the segment of a lesion, while the minimal lumen area (MLA) was the area of the smallest lumen in the segment of a lesion. The reference vessels_(CAE)_ were those with a normal lumen closest to CAE, divided into proximal and distal reference vessels_(CAE)_; The reference vessels_(stenosis)_ were those with a normal lumen closest to the smallest lumen, divided into proximal and distal vessels; the mean reference vessel diameter was the average diameter of the proximal and distal reference vessels; the mean reference vessel area was defined as the average area of the proximal and distal reference vessels; and percent diameter stenosis (%DS) was calculated as (mean reference vessel diameter_(stenosis)_ − minimal lumen diameter)/(mean reference vessel diameter_(stenosis)_) × 100%. Percent area stenosis (%AS) was calculated as (mean reference vessel area_(stenosis)_ − minimal lumen area)/(reference vessel area_(stenosis)_) × 100%.

With regards to image coordinates and distances, we considered the distance between the plaque and CAE as the distance from the frame of the plaque to the nearer edge of the ectasia. The CAE was considered the point of origin. The distance between the proximal plaque and the CAE was recorded as positive, whereas the distance between the distal plaque and the CAE was recorded as negative.

### Statistical analysis

SPSS 25.0 software (SPSS Inc., USA) was used for statistical analysis. After the Kolmogorov–Smirnov test of data distribution, Mean(SD) was used for continuous variables with a normal distribution, while for non-normal variables, continuous variables were expressed as median (Q1, Q3). One-way analysis of variance (ANOVA) was used for comparisons of continuous data with a normal distribution among multiple groups. For non-normal variables, we used nonparametric tests. The Kruskal–Wallis test was used to compare continuous data among groups. Categorical data were presented in the form of counts (proportions) and compared using chi-squared or nonparametric statistics. The primary outcome was MACCEs at 1 year which was defined as a composite of all-cause mortality, non-fatal MI, stroke, repeat revascularization, and hospital admission due to ischemia. Logistic generalized estimating equations (GEE) models were used to calculate odds for the occurrence of main adverse cardiovascular and cerebrovascular events (MACCEs). Two-tailed *P* < 0.05 were considered statistically significant. Results were presented as adjusted odds ratios (aORs) along with 95% confidence intervals (CI). For comparison analysis, the Kruskal–Wallis test with Bonferroni adjustment was used to perform pairwise comparisons between any two groups to compensate for the multiple comparison tests. Multiple comparisons among three groups were performed, for each statistical test, we performed two-sided hypothesis testing with a Bonferroni-corrected alpha of 0.05/3 = 0.017.

## Results

### Baseline clinical characteristics

A total of 286 patients and 344 coronary arteries with CAE were included in this study. The median age of all patients was 57 years and 82.87% of whom were male. The baseline characteristics were summarized in Table 1. Of the 286 patients, 177 suffered MI, and 72 of whom had STEMI. 88/286 (30.77%) patients had multi-vessel CAE. The laboratory data of all patients were also showed in Table 1.

**Table 1:** Baseline clinical characteristics

	Patients with CAE (n = 286)
Age, mean (SD), (years)	57.34 (12.3)
Male, count (%)	237 (82.9)
Hypertension, count (%)	142 (49.7)
Diabetes, count (%)	63 (22.0)
Current smoking, count (%)	147 (51.4)
Prior MI, count (%)	101(35.3)
Hyperlipidemia, count (%)	130 (45.5)
Clinical presentation, count (%)	
Stable angina	6 (2.1)
Unstable angina	103 (36.0)
STEMI	105 (36.7)
NSTEMI	72 (25.2)
Laboratory data	
CK-peak, median (Q1,Q3), (μ/L)	218.00 (88.0–1317.0)
CKMB-peak, median (Q1,Q3), (μ/L)	10.70 (1.00–126.60)
TnI-peak, median (Q1,Q3), (μg/L)	4.71 (0.03–46.21)
hs-CRP, median (Q1,Q3), (mg/L)	3.17(1.37–7.42)
TC, mean (SD), (mmol/L)	4.24 (1.11)
TG, mean (Q1,Q3), (mmol/L)	1.44(1.07–1.95)
HDL, mean (Q1,Q3), (mmol/L)	1.08 (0.93–1.28)
LDL, mean (SD), (mmol/L)	2.78 (1.00)
Creatinine, median (Q1,Q3), (μmol/L)	82.00 (72.00–94.00)
Glycated hemoglobin, mean (Q1, Q3), (%)	5.90 (5.60–7.00)
Multivessel, count (%)	88 (30.8)

Values are mean ± SD, n (%), or median (Q1, Q3). *MI* myocardial infarction; *NSTEMI* non-ST-segment elevation myocardial infarction; *STEMI* ST-segment elevation myocardial infarction, *CK* creatine kinase; *CKMB* MB isoenzyme of creatine kinase; *TNI* troponin I; *hs-CRP* high-sensitivity C-reactive protein; *TC* total cholesterol; *TG* triglyceride; *HDL* high-density lipoprotein; *LDL* low-density lipop

Values are mean ± SD, n (%), or median (IQR). *MI* myocardial infarction; *NSTEMI* non-ST-segment elevation myocardial infarction; *STEMI* ST-segment elevation myocardial infarction; *CK* creatine kinase; *CKMB* MB isoenzyme of creatine kinase; *TNI* troponin I; *hs-CRP* high-sensitivity C-reactive protein; *TC* total cholesterol; *TG* triglyceride; *HDL* high-density lipoprotein; *LDL* low-density lipoprotein

### Angiographic findings

**Fig. 2:**
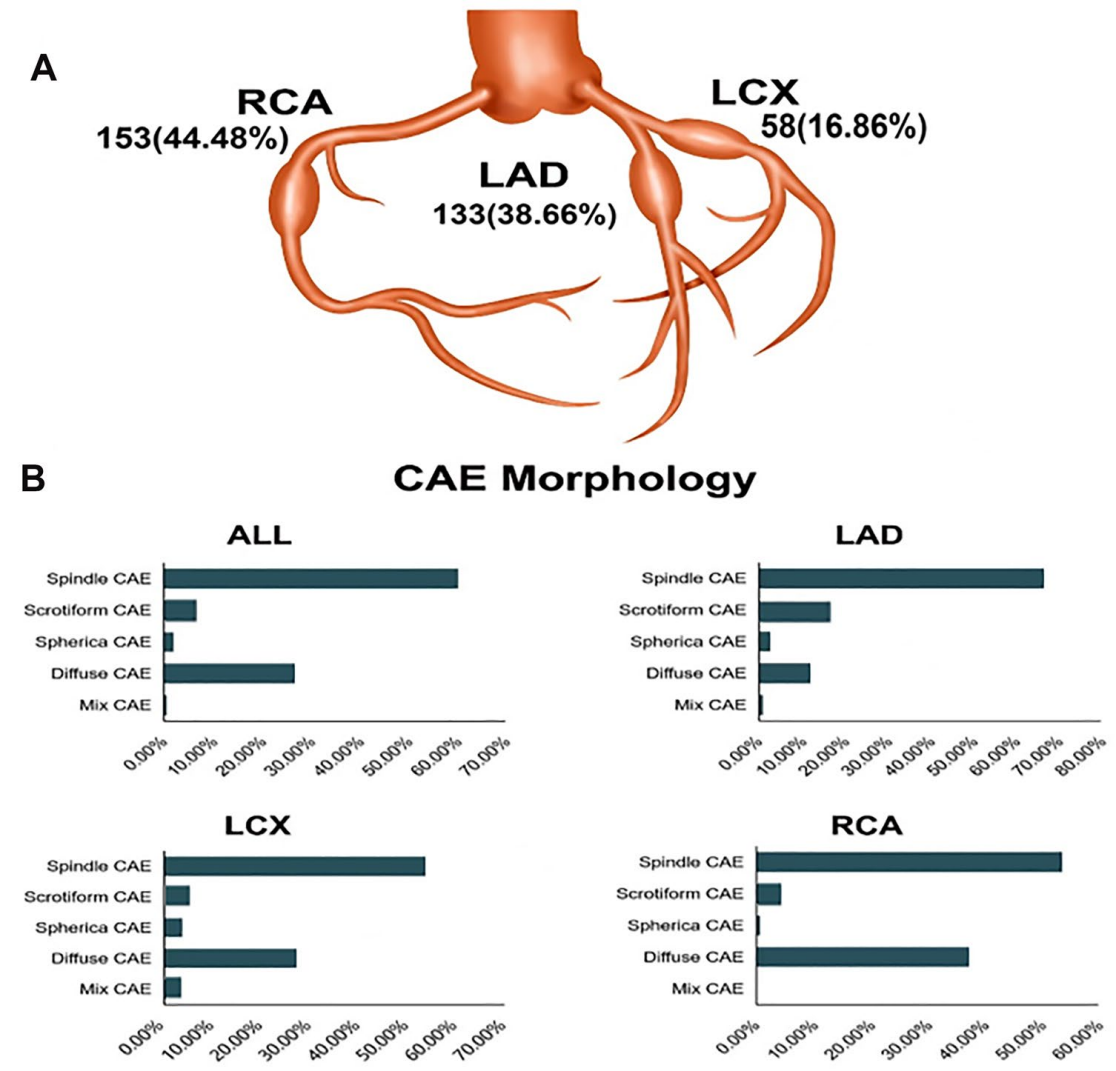
Vascular distribution and morphological types of CAE. **A** Distribution of CAEs. CAE was more likely to appear on the RCA, followed by the LAD artery. **B** Morphological distribution of CAEs. The spindle-shaped CAE was the most common type of CAEs. The ratio of CAE morphology in each vessel was also significantly different. Diffuse CAE most frequently occurred in the RCA, accounting for 44.48% of the vessels. The scrotiform CAE occurrence was significantly higher in the LAD artery than in the other two vessels. *CAE* coronary artery ectasia; *RCA* right coronary artery; *LAD* left anterior descending

Right coronary artery (RCA) lesions were the most common, accounting for 44.48% (n = 153) of CAEs (Fig. 2A). CAE was more likely to occur in the proximal segment (n = 213) of the three coronary arteries on coronary angiography (Table 2). 75.19% (100/133), 72.41% (42/58), and 46.41% (71/153) of the dilatations were occurred in the proximal segment of the left anterior descending (LAD) artery, left circumflex (LCX) artery and RCA and the incidence of CAE in RCA was low (*P* < 0.001).

**Table 2:** Angiographic findings

	LAD (133)	LCX (58)	RCA (153)	*P* < 0.017
CAE lesion site, count (%)				
Proximal CAE segment	100 (75.19)	42 (72.41)	71 (46.41)	a, b, c
Mid segment	31 (23.31)	0 (0)	55 (35.95)	
Distal segment	2 (1.50)	16 (27.59)	27 (17.65)	
TIMI flow, count (%)				
0–1	27 (20.30)	10 (17.34)	38 (24.83)	–
2–3	106 (79.70)	48 (82.75)	115 (75.16)	
CAE morphology, count (%)				
Spindle	91 (68.42)	33 (56.90)	84 (54.90)	b
Scrotiform	19 (14.29)	3(5.17)	5 (3.27)	
Spherica	3 (2.26)	2 (3.45)	2 (1.31)	
Diffuse	18 (13.53)	18 (31.03)	62(40.52)	
Mix	2 (1.50)	2 (3.45)	0	
Culprit vessel, count (%)	101 (75.94)	28 (48.28)	87 (56.86)	a, b
Tortuosity, count (%)	44 (33.08)	21 (36.21)	33 (21.57)	–
Collateral > 2, count (%)	50 (37.59)	30 (51.72)	51 (33.33)	c
Collateral RENTROP, count (%)				
0–1	88 (66.17)	33(56.90)	102 (66.67)	–
2–3	45 (33.83)	25 (43.10)	51 (33.33)	
QCA data, median (IQ1, Q3)				
Minimum lesion diameter, (mm)	1.85 (1.38–2.31)	1.77 (1.33–2.31)	1.99 (1.43–2.60)	–
Maximum lesion diameter, (mm)	4.45 (3.75–5.00)	4.41 (3.81–5.28)	4.52 (4.03–5.22)	–
Diameter stenosis rate, (%)	25.00 (4.00–43.00)	19.00 (12.00–46.00)	31.00 (13.00–50.00)	–

*LAD* left anterior descending artery; *RCA* right coronary artery; *LCX* left circumflex artery; *TIMI* thromboly, *LAD* left anterior descending artery; *RCA* right coronary artery; *LCX* left circumflex artery; *TIMI* thrombolysis in myocardial infarction. Statistically significant difference between: a: Group LAD vs. LCX; b: Group LAD vs. RCA; c: Group LCX vs. RCA

Regarding the shapes of CAE lesions, we found that spindle-shaped CAEs were most common in all coronary vessels, while the diffuse CAEs is more frequently located in the RCA and the LCX arteries and the scrotiform CAEs most occurred in the LAD artery (Fig. 2B). Table 2 showed that coronary tortuosity was observed in 28.49% (98/344) of all coronary arteries. A total of 38.08% (131/344) CAE segments had more than two coronary collateral circulations. CAEs in the LCX artery were more likely to associate with coronary collateral circulation, although there were insignificant differences in RENTROP scores among the vessels (*P* = 0.381). What’s more, based on the qualitative comparative analysis, there existed insignificantly statistical difference in the minimum lesion diameter, maximum lesion diameter, or the diameter stenosis rate in the expansion section among the vessels.

### CAE characteristics of three major coronary vessels assessed by OCT

On basis of OCT images, CAEs were classified by vessel locations (Table 3). In the RCA, the areas of the distal and proximal reference vessels were larger than those in LAD and LCX arteries (*P* < 0.001; *P* < 0.001, respectively). However, there was no difference in the maximum lumen area or diameter of the CAE segments across the LAD, RCA, and LCX arteries. The minimum lumen diameter of the CAE segment was greater in the RCA than in the LAD artery (*P* = 0.001); furthermore, the average length of CAE segments in the RCA was greater than that of the other two vessels (*P* < 0.001). The CAE shape index of the RCA was greater than 2, which was significantly different from that of the other two vessels (*P* < 0.001). There was no significant difference in the area, diameter, or AS% of the smallest lumen evaluated by OCT.

**Table 3:** CAE characteristics of three major coronary vessels by OCT

	LAD (110)	LCX (51)	RCA (134)	*P* < 0.017
Distal Reference Vessel (CAE), median (Q1, Q3)				
Area, (mm^2^)	6.13 (4.94–8.08)	7.55 (4.86–9.41)	8.27 (6.68–10.64)	< 0.001
Mean lumen diameter, (mm)	2.79 (2.49–3.20)	3.09 (2.48–3.45)	3.24 (2.91–3.67)	< 0.001
Proximal reference vessel (CAE), median (Q1, Q3)				
Area, (mm^2^)	7.95 (6.37–11.34)	9.28 (7.09–12.35)	10.14 (7.99–13.52)	< 0.001
Mean lumen diameter, (mm)	3.18 (2.84–3.79)	3.41 (3.00–3.95)	3.55 (3.18–4.14)	< 0.001
CAE, median (Q1, Q3)				
Length, (mm)	9.60 (5.80–17.90)	8.25 (5.10–20.80)	13.80 (7.90–25.60)	< 0.001
Index	1.54 (0.99–2.56)	1.36 (0.83–3.11)	2.14 (1.35–4.36)	< 0.001
Area, (mm^2^)	17.23 (12.74–21.03)	18.28 (13.99–23.68)	18.96 (15.28–23.91)	0.013
Mean lumen diameter, (mm)	4.65 (4.01–5.15)	4.81 (4.22–5.47)	4.90 (4.40–5.50)	0.008
Max lumen diameter, (mm)	5.17 (4.35–5.70)	5.24 (4.63–6.05)	5.31 (4.83–6.00)	0.076
Min lumen diameter, (mm)	4.10 (3.59–4.62)	4.17 (3.67–4.78)	4.43 (3.95–4.97)	0.001
The smallest lumen, median (Q1, Q3)				
Area, (mm^2^)	2.40 (1.56–3.90)	3.16 (1.52–5.61)	3.05 (1.54–6.29)	0.055
Diameter, (mm)	1.74 (1.40–2.20)	1.98 (1.38–2.65)	1.96 (1.39–2.81)	0.055
AS%	66.30 (49.25–79.75)	57.25 (35.53–79.00)	63.15 (40.93–82.78)	0.188

The symbol * stands for inter group comparison. Statistically significant difference between: a: Group LAD vs. LCX; b: Group LAD vs. RCA; c: Group LCX vs. RCA

### Plaque characteristics of three major coronary vessels by OCT

We found 329 (95.64%) CAE vessels with plaques. No significant differences in plaque occurrence were observed among the three vessels (Table 4); LAD was more prone to developing plaque rupture and calcified nodules than the other vessels (*P* = 0.013 and *P* = 0.016). By comparing the properties of lipid plaques and calcified plaques, there did not exist significant differences among the three branches (all *P* > 0.05). Since plaques were very common in CAE vessel segments, the characteristics of these plaques did not significantly differ among the three vessels. Table 4 also showed that there was also an insignificant difference in the presence of cholesterol crystals, thin-cap fibroatheroma, microchannels, and macrophages among vessels. Notably, the probability of thrombus in patients with CAE was 45.59% (150/329) in all vessels. Moreover, the smallest lumens are usually associated with plaques rather than thrombus alone.

**Table 4:** Characteristics of plaques in three major coronary vessels by OCT

	LAD (131)	LCX (52)	RCA (146)	*P* < 0.017
Minimum lumen area, median (Q1,Q3), (mm^2^)	2.40 (1.56–3.90)	3.16 (1.52–5.61)	3.05 (1.54–6.29)	0.055
Distance with CAE, median (Q1,Q3), (mm)	-1.00(-4.90–1.90)	0 (-3.10–1.30)	0 (-2.80–0.80)	0.610
Plaque type, count (%)				
Fibrous plaque	59 (45.04)	18 (34.62)	61 (41.78)	–
Lipid plaque	48 (36.64)	24 (46.15)	70 (47.95)	
Fibrocalcific plaque	24 (18.32)	10 (19.23)	15(10.27)	
Lesion type, count (%)				
Rupture	27 (23.68)	5 (9.62)	30 (20.55)	a
Erosion	73 (64.04)	40 (76.92)	94 (64.38)	
CN	14 (12.28)	1 (1.92)	6 (4.11)	
Plaque characteristics				
Lipid plaque, median (Q1,Q3)				
FCT, (mm)	0.08 (0.06–0.09)	0.08 (0.07–0.09)	0.08 (0.06–0.09)	0.745
Mean lipid arc, (°)	265.80 (195.80–295.80)	271.55 (185.40–324.80)	281.95 (216.20–322.40)	0.496
Lipid core length, (mm)	9.90 (6.30–14.00)	6.65 (4.80–12.30)	8.80 (5.80–16.80)	0.175
Lipid index, median (Q1,Q3),	2669.34 (1383.68–3680.32)	1799.05 (1035.84–2816.04)	2353.18 (1191.24–4968.00)	0.188
Lipid-rich plaque, count (%)	54 (84.38)	21 (77.78)	66(80.49)	–
TCFA, count (%)	16 (25.00)	3 (11.11)	27 (32.93)	–
Calcium, count (%)	68 (51.91)	20 (34.46)	58 (39.73)	–
Maximum calcium arc, median (Q1,Q3), (°)	117.25 (79.45–263.90)	114.25 (84.95–184.45)	104.05 (71.30–180.90)	0.596
Maximum calcium thickness, median (Q1, Q3), (mm)	0.70 (0.47–0.89)	0.66 (0.50–0.71)	0.68 (0.53–0.85)	0.567
Calcium length, median (Q1,Q3), (mm)	5.10 (3.05–9.86)	5.70 (3.05–10.25)	4.25 (2.60–8.10)	0.368
Macrophage, count (%)	115 (87.78)	50 (96.15)	136 (93.15)	–
Microchannel, count (%)	39(29.77)	13(25.00)	51 (34.93)	–
Cholesterol crystal, count (%)	60 (45.80)	26 (50.00)	74 (50.68)	–
Thrombus, count (%)	57 (43.51)	15 (28.85)	78(53.42)	–

*CN* Calcified nodules; *TCFA* thin-cap fibroatheroma; *FCT* fibrous cap thickness. The distance between the proximal plaque and the CAE was recorded as positive whereas the distance between the distal plaque and the CAE was recorded as negative. The symbol * stands for inter group comparison. Statistically significant difference between: a: Group LAD vs. LCX; b: Group LAD vs. RCA; c: Group LCX vs. RCA

### Characteristics of plaques according to the relative positions of CAE segments by OCT

**Fig. 3:**
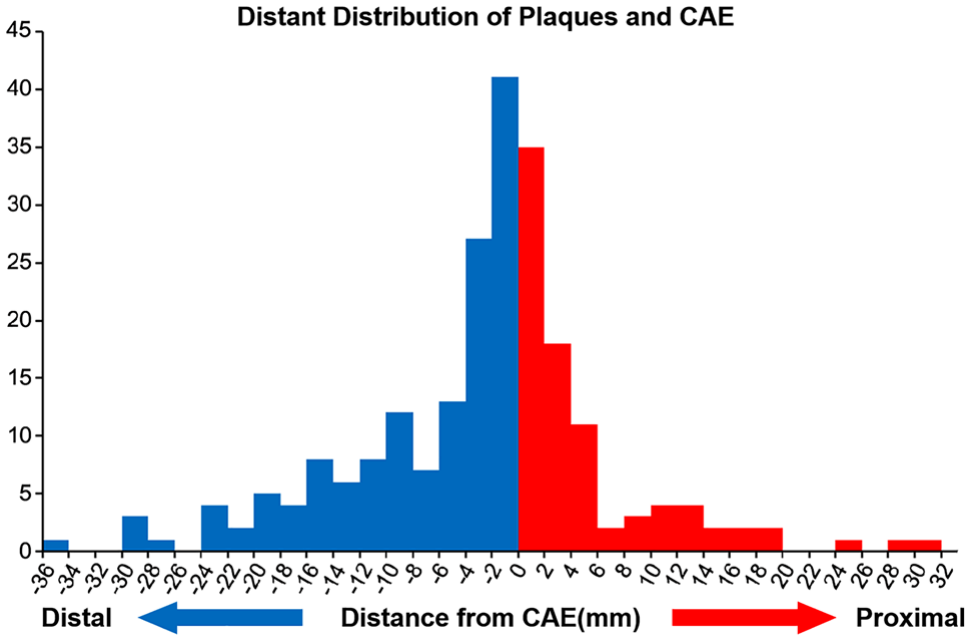
Distance distribution of plaques without and within CAEs. CAE was the zero point. The distance between distal plaques and CAE lesions was longer than that between proximal plaques and CAE lesions. *CAE* coronary artery ectasia

**Fig. 4:**
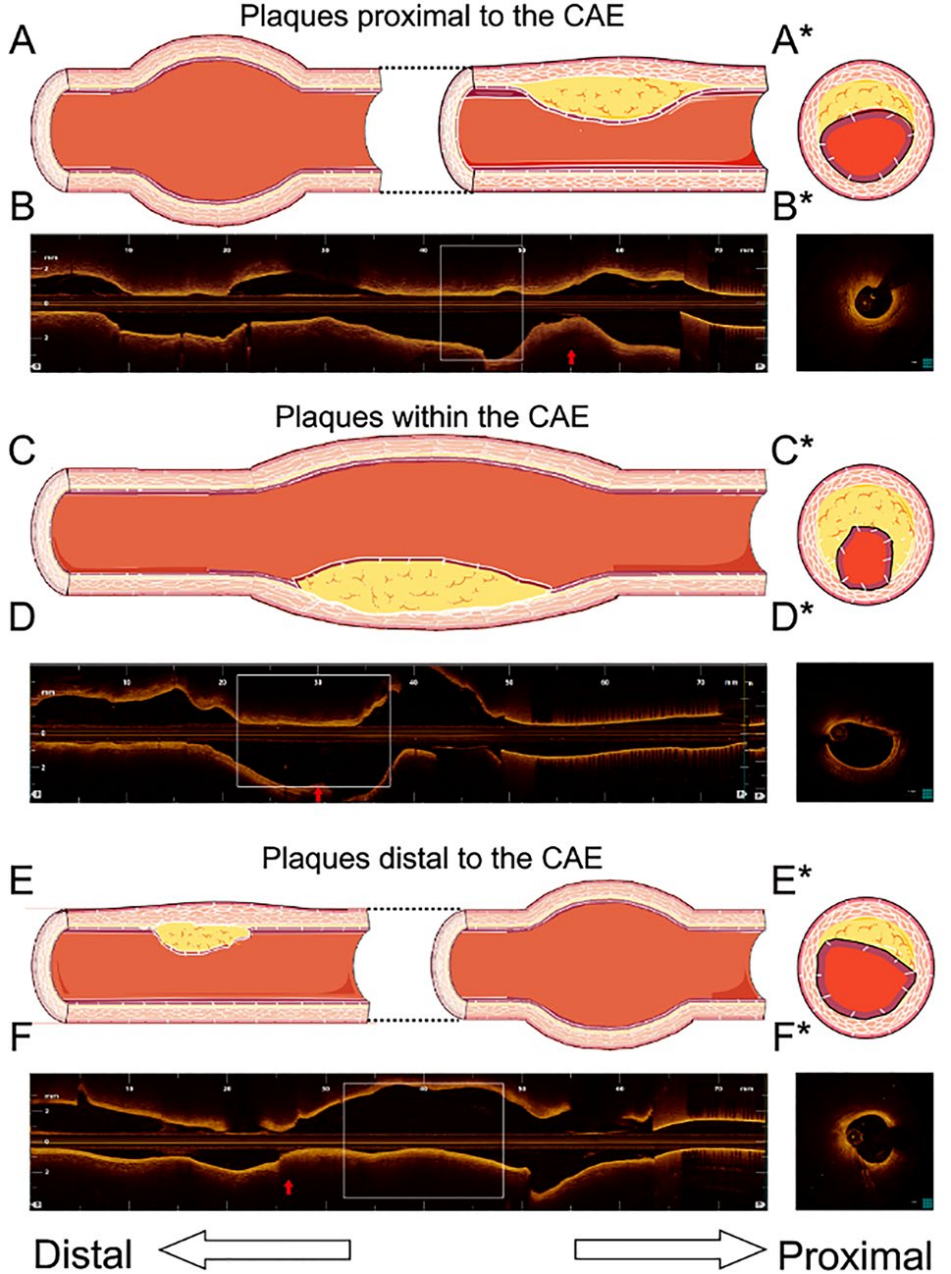
The characteristics of plaques among plaques proximal to the CAE, plaques within the CAE, and plaques distal to the CAE. Plaques within the CAE were more vulnerable. Plaques within the CAE were the longest, compared to other positions (P = 0.002). Plaques within the CAE had the greatest mean lipid angle (P = 0.035). The white square area was the CAE area. Representative angiography images of the three types were listed on the left. The red arrow was pointing to MLA. *was the cross section of the MLA

48.33% (159/329) of plaques were at the distal position of CAE segments. Plaques within the CAE lesion were associated with the largest minimum lumen area (*P* = 0.010). The distance between distal plaques and the CAE lesions was longer than that between the proximal plaques and the CAE lesions (*P* < 0.001). Plaques within the CAE lesions were longer than those in the other positions (*P* < 0.001). Moreover, plaques within the CAE lesions had greater maximum lipid angles (*P* = 0.007) and lipid indexes (*P* = 0.004) than those of plaques in other locations. We also found that the overall incidence of CAE was not influenced by AS%, CAE morphology, or CAE location. We divided plaques into those proximal to, within, and distal to the CAE lesions. Plaques were most likely found within 3.10 mm proximal and 4.25 mm distal to the CAE lesions (Fig. 3). Plaques within CAE lesions had the largest mean lipid angle and the greatest lipid index. Furthermore, plaques within CAE lesions were longer than those on other sites. The mean lipid angle of plaques within CAE lesions was larger than that of plaques in other sites. These findings suggest that plaques within CAE lesions are more vulnerable than those in other locations (Fig. 4). These results suggest the use of optimized lipid-lowering therapy, such as PCSK-9 inhibitors, for patients with plaques within CAE lesions (Table 5).

**Table 5:** Characteristics of plaques according to the relative positions of CAE segments by OCT

	Proximal CAE (110)	Within CAE (60)	Distal CAE (159)	*P* < 0.017
Minimum lumen area, median (IQR), (mm^2^)	2.75 (1.66–6.08)	3.20(1.74–7.31)	2.39 (1.41 to 4.55)	0.010
Distance with CAE, median (IQR), (mm)	3.20(1.60–7.80)	0.00 (0.00–0.00)	− 4.30 (− 11.70 to − 1.80)	< 0.001
Plaque length, median (IQR), (mm)	9.70 (5.70–18.60)	24.60 (16.60–41.65)	9.30 (5.90 to 16.20)	< 0.001
Plaque type, count (%)				
Fibrous plaque	49 (44.55)	16(26.67)	73 (45.91)	c
Lipid plaque	45 (40.91)	36 (60.00)	61 (38.36)	
Fibrocalcific plaque	16 (14.55)	8 (13.33)	25 (15.72)	
Lesion type, count (%)				
Rupture	19 (20.65)	17 (32.08)	26 (16.35)	–
Erosion	66 (71.74)	30 (50.00)	111(76.03)	
CN	7 (7.61)	6 (10.00)	8(5.03)	
Plaque characteristics				
Lipid plaque, median (IQR)				
FCT, (mm)	0.08 (0.06–0.10)	0.07 (0.06–0.09)	0.08 (0.06 to 0.09)	0.334
Mean lipid arc, (°)	255.13 (192.20–301.80)	302.00 (234.20–360.00)	267.60 (181.00 to 303.00)	0.007
Lipid core length, (mm)	9.70(5.80–14.60)	12.40 (6.70–19.20)	8.1 (5.20 to 12.80)	0.009
Lipid index	2522.70 (1368.00–3380.40)	3240.00 (1827.65–6275.70)	2030.10 (875.84 to 3402.24)	0.004
Lipid-rich plaque, count (%)	46 (41.82)	35 (58.33)	61(38.36)	c
TCFA, count (%)	12 (10.91)	15(25.00)	19 (11.95)	a, c
Calcium, count (%)	48 (43.64)	25 (41.67)	73(45.91)	-
Maximum calcium arc, median (IQR), (°)	104.85 (77.55–156.40)	90.60 (61.80–149.60)	117.30 (87.00 to 271.10)	0.141
Maximum calcium thickness, median (IQR), (mm)	0.69 (0.44–0.90)	0.65(0.53–0.97)	0.67(0.49 to 0.81)	0.931
Calcium length, median (IQR), (mm)	5.30 (3.00–10.25)	4.50(3.00–8.10)	5.10 (2.80 to 8.40)	0.921
Macrophage, count (%)	95 (86.36)	54 (90.00)	144(90.57)	–
Microchannel, count (%)	37 (33.64)	18 (30.00)	44 (27.67)	–
Cholesterol crystal, count (%)	60 (45.45)	35 (58.33)	71 (44.65)	–
Thrombus, count (%)	43(39.09)	33 (55.00)	69 (43.40)	–

The symbol * stands for inter group comparison. Statistically significant difference between: a: Group proximal CAE vs. within CAE; b: Group proximal CAE vs. distal CAE; c: Group within CAE vs. RCA

### Outcome measures and risk factor of MACCEs occurrence

According to Table S1, one-year MACCE rates were similar among three groups [LAD 16.5%, LCX 19.0%, and RCA 13.7% (*P* = 0.610)]. The aOR for MACCEs was calculated using a logistic GEE model, adjusted for the plaque length, calcification, location, plaque type, and distance of CAE from plaque. The location of CAE was an independent factor associated with the presence of MACCEs [OR 0.886 (95% CI: 0.790–0.993); *P* = 0.037]. However, insignificance was identified for length, diameter, or size CAE at diagnosis (all *P* > 0.05) (Table S2).

## Discussion

To our knowledge, this is the first study to use OCT to assess the potential differences in the prognostic factors of patients who suffered CAE and the characteristics of CAE-associated plaques among vessels. The main findings of this study were as follows: Firstly, RCA was the most common vessel affected by CAEs, although with a smaller size of CAEs than those in the other two vessels. Secondly, atherosclerotic plaques occurred in nearly all CAE segments, which may indicate a close relationship between CAE and AMI. Most importantly, the location of CAE was an independent factor associated with the presence of MACCEs.

A previous study found that the RCA was involved in 44% of CAE cases, whereas the LCX and LAD accounted for 18% and 38% of all CAE cases, respectively [6]. Our findings were consistent with the previous study, and 44.48% of CAE lesions were found in RCA. However, more than 90% of CAEs were found at major coronary branching sites in the LAD artery in KD patients [12]. Thus, the pathological features obviously differed between patients with atherosclerosis from KD-related CAEs. Specifically, OCT showed that the proximal and distal reference vessels of CAE were significantly different. The reference lumen area and diameter of the RCA were significantly larger than those of the LCX and LAD arteries. However, there was no difference in the maximum lumen area and diameter of the CAE segment among the three vessels. Thus, in the RCA, CAE had the lowest degree of dilation, although the RCA is the most common CAE-affected vessel. Further research is needed to understand the mechanism underlying these differences.

Surprisingly, nearly all vessels in CAE patients were accompanied by atherosclerotic plaques. According to previous studies, CAE was a variation of coronary heart disease (CHD) for the two diseases that have similar features, including risk factors, clinical symptoms and pathological findings [13]. CAE patients in the combination of plaques carries an increased risk of MI due to slower blood flow and coronary thrombosis, typically within the dilated segments. Because of the thrombosis risk associated with CAE, many authors have recommended antiplatelet therapies when diagnosed CAE; however, it’s still controversial. Allowing for the various kinds of underlying etiologies that can be related to CAEs, treatment need to be tailored according to the severity and prognosis associated with each individual case. Moreover, plaques located within CAE were more vulnerable than those located proximal or distal to the CAE lesions, and it seemed that the different plaque positions relative to the CAE lesions were closely related to hemodynamics. One previous study demonstrated that these strong flow vortices by CAE caused low wall shear stress and a high oscillatory shear index, which could make plaques in this area more vulnerable [14]. Based on our findings, we suggested that the hemodynamic changes caused by AS may not necessarily affect CAE, although the hemodynamic changes caused by CAE will affect the properties of atherosclerotic plaques.

Given the high incidence of atherosclerotic plaques in CAE-related arteries, they may be filled with heavy thrombus, and the no-reflow phenomenon and distal embolization after primary PCI were observed at significantly higher rates than those in non-CAE patients. Although one-year MACCE rates were similar among the three groups, the location of CAE was an independent factor associated with the presence of MACCEs. Thus, coronary flow in separate coronary artery may be an active process dependent on function of ectatic coronary arteries which affect the prognosis of different patients.

Our study has some limitations. Firstly, this study was retrospective study in a single-center. A prospective study is needed to validate our results. Second, OCT is not the most sensitive intravascular imaging modality for evaluating CAEs, as the observed depth of OCT is less than that of IVUS. However, the pathological manifestations of CAE are remarkably diverse, such as extensive destruction of musculoelastic elements, elastin fibers and components of the extracellular matrix of the coronary wall [15, 16]. In recent years, little progress has been made in identifying the pathophysiologic background of CAE, advances in improved imaging modalities may enable more accessible diagnosis and evaluation. Specifically, OCT has the advantage of greater image resolution and therefore provides more granular details about vessel wall and plaque characteristics compared to IVUS. Future research using dual imaging modality in combination with fluid dynamics or mechanical stress may provide further information on CAE [17, 18]. Thus, we hypothesized that OCT may provide insights into the evaluation of characteristics of CAE and update on the current knowledge on CAE. Finally, in patients with a TIMI flow grade ≤ 2, culprit lesion morphologies may have been altered by aspiration thrombectomy before OCT imaging.

## Conclusion

OCT allows specific phenotyping and characterization of morphologic and functional impairments of arteries in CAE patients. We found that atherosclerotic plaque has a prevalence of 95.6% among CAE patients undergoing coronary angiography. The characteristics of accompanying plaques were not affected by the location of vessels or the morphology of CAE, but rather by the position of the plaques relative to the CAE lesions. Tailored treatment may be required to improve the prognosis. Although prognostic value of CAE location was found, further investigations were still warranted.

## Supplementary Information

Below is the link to the electronic supplementary material.

Supplementary file1 (DOCX 17 KB)
