# Supplementary material for: Characteristics of coronary artery ectasia and accompanying plaques: an optical coherence tomography study
Source: Int J Cardiovasc Imaging. 2023 Apr 26;39(7):1357–66. doi: 10.1007/s10554-023-02835-9 (PMC10250510; doi:10.1007/s10554-023-02835-9)
Supplement: Supplementary file 1 — Supplementary file1 (DOCX 17 KB) [file 10554_2023_2835_MOESM1_ESM.docx]

1. **Methods** (*Supplementary text online*)

**2.2 Angiography and Analysis**

An independent core laboratory performed angiographic analysis by using a quantitative coronary angiogram analysis program (QCA) (Cardiovascular Angiography Analysis System 5.10, Pie Medical Imaging B.V., Maastricht, the Netherlands). CAE is defined as a diameter of ectasia larger than 1.5-fold that of adjacent normal segments by angiography. The morphology of CAE was determined according to L (the length of CAE, mm) and D (the maximum diameter of CAE, mm) measured by QCA. CAE shape index=L/D ^1^. CAE shape index > 1 is spindle CAE. CAE shape index<1 is a scrotiform CAE. CAE shape index = 1 is a spherical CAE. Diffuse CAE is defined by the L being longer than 50% of the length of the CAE-located coronary artery ^2^. Mixed CAE refers to the presence of two or more kinds of CAEs in the same coronary artery. Coronary tortuosity was defined as two or more coronary artery curves ≥ 75° or at least one curve ≥ 90° ^3^. The coronary collateral circulation was assessed according to RENTROP ^4^.

**References**

1. Fan T, Zhou Z, Fang W, Wang W, Xu L, Huo Y. Morphometry and hemodynamics of coronary artery aneurysms caused by atherosclerosis. *Atherosclerosis* 2019;**284**:187-93. Doi:10.1016/j.atherosclerosis.2019.03.001.

2. Kruger D, Stierle U, Herrmann G, Simon R, Sheikhzadeh A. Exercise-induced myocardial ischemia in isolated coronary artery ectasias and aneurysms ("dilated coronopathy"). *J Am Coll Cardiol* 1999;**34**:1461-70. Doi:10.1016/s0735-1097(99)00375-7.

3. Hutchins GM, Bulkley BH, Miner MM, Boitnott JK. Correlation of age and heart weight with tortuosity and caliber of normal human coronary arteries. *Am Heart J* 1977;**94**:196-202. Doi:10.1016/s0002-8703(77)80280-9.

4. Rentrop KP, Cohen M, Blanke H, Phillips RA. Changes in collateral channel filling immediately after controlled coronary artery occlusion by an angioplasty balloon in human subjects. *J Am Coll Cardiol* 1985;**5**:587-92. Doi:10.1016/s0735-1097(85)80380-6.
